# Immunogenic cell death-related biomarkers in heart failure probed by transcriptome and single-cell sequencing

**DOI:** 10.3389/fimmu.2025.1560903

**Published:** 2025-06-24

**Authors:** Haoyue Wang, Dongdong Wu, Gangfei Han, Jingjing Yan, Zehui Wang, Xing He, Yuxiang Chen, Yan Wang, Qinghua Han

**Affiliations:** ^1^ Department of Cardiology, The First Hospital of Shanxi Medical University, Taiyuan, Shanxi, China; ^2^ The First Clinical Medical College, Shanxi Medical University, Taiyuan, Shanxi, China; ^3^ Department of Hepatobiliary Surgery and Liver Transplantation Center, The First Hospital of Shanxi Medical University, Taiyuan, Shanxi, China; ^4^ Department of Health Statistics, School of Public Health, Shanxi Provincial Key Laboratory of Major Diseases Risk Assessment, Shanxi Medical University, Taiyuan, Shanxi, China; ^5^ College of Basic Medicine, Shanxi Medical University, Taiyuan, Shanxi, China; ^6^ Key laboratory of Cellular Physiology at Shanxi Medical University, Ministry of Education, Taiyuan, Shanxi, China; ^7^ Shanxi Innovation Center for Integrated Management of Hypertension, Hyperlipidemia and Hyperglycemia Correlated with Cardiovascular and Cerebrovascular Diseases, Taiyuan, Shanxi, China

**Keywords:** immunogenic cell death, heart failure, biomarker, single-cell RNA sequencing analysis, monocytes and macrophages

## Abstract

**Background:**

Heart failure (HF) represents the terminal stage of various cardiovascular disorders, with immunogenic cell death (ICD) potentially influencing HF progression through modulation of immune cell activity. This study aimed to identify ICD-associated biomarkers in patients with HF and explore their underlying mechanisms.

**Methods:**

Data from GSE57338, GSE3586 and GSE5406 were retrieved from the Gene Expression Omnibus (GEO) database. Differential expression analysis and weighted gene co-expression network analysis (WGCNA) were employed to identify candidate genes, followed by enrichment analysis and Protein-Protein Interaction (PPI) network construction. Candidate biomarkers were selected using two machine learning approaches and validated for expression levels, with receiver operating characteristic (ROC) curve analysis determining the final biomarkers. A nomogram model was built based on the biomarkers, followed by molecular regulatory network analysis, gene set enrichment analysis (GSEA), immune infiltration assessment, and drug prediction. Additionally, key cells were selected for pseudo-time and cell communication analysis using the GSE183852 dataset. Next, pseudotemporal analysis was also performed on key cell subpopulations. Real-time quantitative PCR (RT-qPCR) was employed to validate the biomarkers.

**Results:**

Three biomarkers, CD163, FPR1, and VSIG4, were identified as having significant diagnostic value for HF. GSEA revealed their enrichment in ribosomal and immune cell-related pathways. These biomarkers were notably correlated with CD8 T cells and M2 macrophages. Carbachol and etynodiol were predicted to interact with all three biomarkers. Single-cell RNA sequencing identified nine cell types, with expression of the biomarkers confined to monocytes and macrophages. Strong cell communication was observed between these cell types and fibroblasts. Expression of CD163 and VSIG4 decreased over time in monocytes and macrophages, whereas FPR1 showed an upward trend. In addition, the expression levels of CD163 and VSIG4 increased in subpopulations of monocytes and macrophages, whereas FPR1 showed a decreasing trend. RT-qPCR results confirmed significant down-regulation of CD163, FPR1, and VSIG4 in patients with HF and animal models.

**Conclusions:**

This study identified and validated three ICD-related biomarkers in HF—CD163, FPR1, and VSIG4—offering a novel theoretical foundation for the clinical diagnosis and treatment of HF.

## 1 Introduction

Heart failure (HF), the terminal stage of various cardiovascular diseases, affects approximately 56.2 million people worldwide (1, 2). Despite lifestyle changes and advances in medical care that have stabilized age-adjusted incidence rates, the prevalence and mortality rates of HF remain high, highlighting the need for further research to identify improved management strategies (3). Although HF was once considered non-immune-mediated, recent studies have demonstrated the involvement of the immune system in its pathophysiology, and clinical trials on immune modulation therapy for HF have been conducted (4). Consequently, modulating immune responses to maintain stability may serve as a promising strategy to delay HF progression.

Immunogenic cell death (ICD), a unique form of regulated cell death that occurs as a downstream effect of tumor-specific immune responses, has been extensively studied in cancer immunotherapy (5, 6), with emerging research in cardiovascular diseases. Endothelial cell ICD in atherosclerosis has been linked to the initiation of adaptive immune responses, sustaining chronic inflammation within plaques (7). In coronary artery disease, stratification based on ICD-related genes (IRGs) enables the development of risk models and immune subtypes that facilitate treatment decisions (8). Moreover, ICD has been explored as a diagnostic tool for ischemic stroke in elderly women, identifying key biomarkers for diagnosis (9). However, the mechanisms underlying ICD in HF remain unexplored.

This study utilized machine learning techniques to identify ICD biomarkers in HF, followed by immune infiltration analysis, targeted drug prediction, gene set enrichment analysis (GSEA), single-cell data clustering and annotation, cell communication analysis, and pseudotime analysis. The findings revealed the functional and potential molecular mechanisms of these biomarkers at both the transcriptomic and cellular levels, providing a novel theoretical framework for the clinical diagnosis and treatment of HF.

## 2 Materials and methods

### 2.1 Data collection

RNA data from GSE57338 (sequencing platform: GPL11532) was obtained from the Gene Expression Omnibus (GEO) database (https://www.ncbi.nlm.nih.gov/geo/), comprising 136 normal left ventricular tissue samples and 177 left ventricular tissue samples from patients with HF (10). Additionally, RNA data from GSE3586 (sequencing platform: GPL3050) was downloaded, containing 15 normal left ventricular tissue samples and 13 left ventricular tissue samples from patients with HF (11). Moreover, the GSE5406 dataset contained 16 normal and 194 HF patients’ heart tissue samples. The data were obtained from the GPL96 platform using chip sequencing technology, mainly for biomarkers expression validation. The single-cell dataset GSE183852 was retrieved from the GEO website (sequencing platform: GPL24676), including heart tissue samples from 5 patients with HF and 2 normal heart tissue samples (12). A total of 34 ICD-associated genes were obtained from the literature (13) (**Additional file 1**).

### 2.2 Differential expression analysis

Differential expression analysis was conducted using the R package “limma” (v 3.58.1) (14), applying the screening criteria of *|log_2_fold change (FC)|* > 0.5 and *P* < 0.05 to compare HF and control samples in the GSE57338 dataset. Volcano plots of the differentially expressed gene (DEGs) were visualized using the R package “ggplot2” (v 3.4.1) (15), highlighting the top 10 up- and down-regulated DEGs. Heatmaps of the top 10 DEGs were generated using the R package “ComplexHeatmap” (v 2.4.0) (16).

### 2.3 Weighted gene co-expression networks analysis

To calculate the single-sample gene set enrichment analysis (ssGSEA) scores for ICD-related genes across 313 samples, the ssGSEA algorithm from the R package “GSVA” (v 1.46.0) (17) was applied, and box plots were created using “ggplot2” (v 3.4.1).

WGCNA was performed on the GSE57338 dataset using the R package “WGCNA” (v 1.72.5) (18), with ssGSEA scores as the feature. Initial clustering of samples identified and excluded abnormal samples. The soft threshold (power) was determined based on an *R^2^* > 0.85 and mean connectivity = 0. The dynamic tree cutting algorithm, with a minimum gene number of 50 per module and a module merging threshold of 0.3, was applied to define gene modules. Genes were color-coded, and the “grey” module (containing unclassified genes) was excluded. Pearson correlation coefficients were calculated between the modules and ssGSEA scores, with a heatmap generated to highlight modules with significant correlation (*|cor|* > 0.5, *P* < 0.05). Genes within these modules were identified as key module genes.

### 2.4 Enrichment analysis of candidate genes and protein-protein interactions network analysis

The R package “ggvenn” (v 1.7.3) (19) was employed to identify the intersection between DEGs and key module genes, resulting in the selection of candidate genes. These genes were then converted from SYMBOL to ENTREZID using the human genome database org.Hs.eg.db (v 3.18.0) (20). Candidate genes underwent Gene Ontology (GO) and Kyoto Encyclopedia of Genes and Genomes (KEGG) functional enrichment analysis with the R package “ClusterProfiler” (v 3.16.0) (21), with a threshold of *P* < 0.05. To construct PPI networks, candidate genes were analyzed using the search tool for the retrieval of interacting genes (STRING) database (https://string-db.org) with a confidence score of 0.4. PPI networks were then visualized with Cytoscape (v 3.10.0) (22). The Cytohubba plugin in Cytoscape (v 3.10.0) was utilized to rank candidate genes using six algorithms: Maximum Connectivity Component (MCC), Minimum Network Connectivity (MNC), Degree of Minimum Network Connectivity (DMNC), Degree, Closeness, and Betweenness. Based on the ranking results, the top 20 genes from each algorithm were extracted, and their intersection was used to identify the final candidate key genes. UpSet plots were generated using the R package (v 1.4.0) (23).

### 2.5 Screening candidate biomarkers by machine learning

Candidate key genes were further screened based on sample grouping information from GSE57338 using the support vector machine-recursive feature elimination (SVM-RFE) algorithm (10-fold cross validation) (v 4.1.4) (24) to obtain feature genes. The R package “randomForest” (v 3.2.2) (25) was used for random forest algorithm analysis of the feature genes, incorporating sample grouping information from GSE57338. A total of 500 decision trees were computed using the randomForest function, and the MeanDecreaseGini values for each feature gene were visualized in a bar chart. The median of the MeanDecreaseGini values (MeanDecreaseGini measures the effect of each variable on the heterogeneity of observations at each node in the classification tree, thus assessing the importance of the variable. The larger the value, the higher the importance of the variable) was calculated, and genes with values above the median were selected as candidate biomarkers. Correlation analysis of the candidate biomarkers was performed using the R package “corrplot” (v 0.92) (26), with thresholds of |cor| > 0.3 and *P* < 0.05.

### 2.6 Expression validation of candidate biomarkers

Expression differences of candidate biomarkers between HF and normal samples were analyzed using the grouping information from GSE3586 and GSE57338, with a threshold of *P* < 0.05. Box plots were constructed using the R package “ggplot2” (v 3.4.1). Candidate biomarkers showing differential expression between groups and consistent trends across both datasets were selected for receiver operating characteristic (ROC) analysis. ROC curves for candidate biomarkers were generated using the R package “pROC” (v 1.18.0) (27), and the area under the curve (AUC) was calculated, with biomarkers defined as those having an AUC > 0.7. To validate biomarkers expression, differential expression analysis was performed in the GSE5406 dataset.

### 2.7 Construction of a nomogram

In the GSE57338 dataset, a nomogram was constructed using the R package “rms” (v 5.1.4) (28) to evaluate the risk of developing HF, based on the expression of identified biomarkers. The predictive performance of the nomogram was assessed by plotting the ROC curve with the R package “pROC” (v 1.18.0).

### 2.8 Gene set enrichment analysis

Spearman correlation analysis was performed between each biomarker and the remaining genes across all GSE57338 samples using the R package “psych” (v 2.2.9) (29), generating correlation coefficients. Genes were then ranked according to these coefficients, yielding gene lists associated with each biomarker. GSEA was performed using the sorted results and the R package “ClusterProfiler” (v 3.16.0), with “c2.kegg.v7.4.symbols.gmt” and “c5.go.v7.4.symbols.gmt” from the Molecular Signatures Database (MSigDB, https://www.gsea-msigdb.org/gsea/msigdb/index.jsp) as reference gene sets. The top 5 most significant signaling pathways were visualized using the enrichplot package (*P* < 0.05 and |Normalized Enrichment Score (NES)| > 1) (v 1.18.3) (20).

### 2.9 Immune infiltration analysis

The CIBERSORT algorithm (v 1.03) (30) was employed to calculate the relative abundance of 22 immune cell types (31) in HF and normal samples from the GSE57338 dataset. Immune cells with a result of 0 were excluded. Differential immune cells (*P* < 0.05) were identified, and box plots were constructed for visualization. Spearman correlation analysis was used to assess the relationships among differential immune cells and between biomarkers and immune cells (|cor| > 0.3 and *P* < 0.05). A correlation matrix was created using the R package “corrplot” (v 0.92) (26), and a heatmap was plotted using the R package “pheatmap” (v 1.0.12) (32).

### 2.10 Regulatory network analysis

MiRNAs targeting the biomarkers were predicted using the microRNA database (miRDB, http://mirdb.org) and the starBase database (http://starbase.sysu.edu.cn/), and the intersection of miRNAs from both databases was extracted. Based on these predictions, a miRNA-biomarker network was constructed using Cytoscape (v 3.10.0). Transcription factors (TFs) related to the biomarkers were identified using the TRRUST database (http://www.grnpedia.org/trrust/), while the disease signatures database (DSigDB, https://www.dsigdb.org/) was used to identify drugs targeting the biomarkers. A biomarker-drug network was then created and visualized.

### 2.11 Single-cell RNA sequencing analysis

The single-cell RNA sequencing data from GSE183852 were processed into Seurat objects using the R package “Seurat” (v 4.4.0) (33). Quality control was performed by applying the following parameters: 200 < nFeature_RNA < 4,000, nCount_RNA < 10,000, and Mt < 10%. Genes covered by fewer than three cells were removed. Hypervariable genes were selected using variance stabilization transformation (vst), and the highly variable genes (HVGs) were retained for further analysis. The LabelPoints function was applied to identify the top 10 most variable genes, and the Scale Data function was used for normalization. Principal component analysis (PCA) was performed on the HVGs for dimensionality reduction. The p-value for PCs 1 to 15 was calculated using the Jackstraw function, and variance drop values for PCs were computed using the Elbowplot function. Based on the elbow plot, appropriate PCs were selected for subsequent analysis (*P* < 0.05). Uniform Manifold Approximation and Projection (UMAP) clustering analysis was applied to identify cell clusters (resolution = 0.5). Cellular annotation was performed according to the literature (12). The Dotplot function was used to visualize the expression of the three biomarkers in the cells, and cells expressing all three biomarkers were selected as key cells. Enrichment analysis for each cell subtype was conducted using the analyze_sc_clusters function from the R package “ReactomeGSA” (v 1.12.0) (34). The pathways function was used to extract enrichment results, and a heatmap displayed the top ten enriched pathways in each cell subtype. Cell subtype interactions were explored using the R package “CellChat” (v 1.6.1) (35) to conduct communication analysis. Trajectory differentiation of key cell clusters was simulated using the R package “Moncle” (v 2.30.1) (36). The dynamic trend of biomarker expression during cell differentiation was plotted using the plot_pseudo-time_heatmap function. Next, the marker genes of key cell subpopulations were selected for annotation based on the CellMarker 2.0 database (https://ngdc.cncb.ac.cn/databasecommons/database/id/6110), and the final key cell subpopulations were identified based on the specific expression of these genes in different clusters. To further explore the expression dynamics and temporal trajectories of biomarkers in the key cells, the annotated key cell subpopulations were analyzed by the proposed timeline trajectory analysis. Using the R package Monocle2 (v 2.24.1) (37), the distribution of biomarkers in each key cell subtype was projected onto a root and multiple branches, a single-cell trajectory map was constructed, and the dynamic trend of biomarker expression during cell differentiation was plotted. Subsequently, in order to analyze the relationship between differentiation states and subtypes of key cells, stacked maps of cell subpopulations in different differentiation states were drawn. Based on the subtype annotation results, the proportions of cell types under different groupings were first visualized. Wilcoxon test. Finally, the differences in the expression of NOS2, TNF, ARG1, and MRC1 genes in Monocyte&Macrophage between HF and control samples were analyzed and statistically analyzed using the Wilcoxon test.

### 2.12 Human Subjects and Extraction of PBMC

Patients with HF admitted to the First Hospital of Shanxi Medical University were selected as the HF group, and a control group was matched with the HF group based on age, gender, and other underlying diseases besides HF. Based on the expression of biomarkers obtained through bioinformatics, the sample size was calculated using PASS.15, resulting in a total of 15 pairs of samples. In the morning of the second day after admission, venous blood was collected into EDTA tubes, and peripheral blood lymphocytes were isolated within 2 hours using human peripheral blood lymphocyte separation liquid (Solarbio, China). The trial protocol was approved by the Scientific Research Ethics Review Committee of the First Hospital of Shanxi Medical University (NO. KYLL-2024-236), and all patients provided written informed consent.

### 2.13 Animal model (echocardiography)

SSPF-grade male Sprague-Dawley rats (180–200 g, 6–8 weeks old) were used to establish a chronic HF model (38). HF was induced by permanently ligating the left coronary artery in rats, while sham-operated rats underwent the same surgical procedure without artery ligation. Six weeks post-ligation, high-resolution echocardiography was performed using the Vevo 770 system (Visualsonics) with a 40 MHz RMV 704 scanhead to assess cardiac function. Rats with an ejection fraction (EF) < 40% were considered to have successfully developed HF, and those that did not develop HF were excluded. After completing echocardiography, the animals were euthanized, and tissues were collected for analysis. The experimental protocol was approved by the Animal Experimental Center Ethics Committee of Beijing Yongxinkangtai Science and Technology Development Co., Ltd. (NO. YXKT2024L010).

### 2.14 Staining

Hearts were fixed in 4% paraformaldehyde at room temperature for 48 hours, followed by dehydration and embedding. The samples were sectioned at 5μm thickness, dewaxed, rehydrated, and stained with Hematoxylin and Eosin (HE) and Masson stains. For IHC staining, primary antibodies targeting CD163 (1:200, Selleck, F1548) was incubated overnight at 4 °C. Then, second antibody was incubated at 37°C for 1 hour. Chromogen development was accomplished with DAB. Images were captured under a microscope (Olympus, Japan).

### 2.15 Real-time quantitative PCR

Following tissue homogenization, total RNA was extracted using Trizol (Thermo Fisher Scientific, USA). cDNA synthesis was carried out using PrimeScript RT Master Mix (Takara, Japan) according to the manufacturer’s protocol. Real-time quantitative PCR (qPCR) analysis was performed with SYBR Green Master Mix (DBI Bioscience, Germany) on a QuantStudio3 real-time PCR instrument (Thermo Fisher Scientific, USA), with GAPDH as an internal control. Relative mRNA expression levels were quantified using the 2^-ΔΔCt^ method. Primer sequences are provided in **Table 1**.

**Table 1:** Primer sequences for quantitative real-time PCR.

Species	Target gene		Primer sequence (5’to3’)
Human	VSIG4	Forward	AAGCAACATCTACAGTGAAGCAGTC
		Reverse	ATGATGAGGATGATGGCAAAGACAG
	FPR1	Forward	AGTGGACATCAACTTGTTCGGAAG
		Reverse	ACGGTGCGGTGGTTCTGG
	CD163	Forward	ACAATGAAGATGCTGGCGTGAC
		Reverse	TCTCTGAATCTCCACCTCAACTGTC
	GAPDH	Forward	CGTATCGGACGCCTGGTT
		Reverse	AGGTCAATGAAGGGGTCGTT
Rat	VSIG4	Forward	AGCTGCCGATCTTTGCCATAATC
		Reverse	TCCTGCTCACCTCATAGACATACTC
	FPR1	Forward	CCGTGAACACTTGAGGAACATACC
		Reverse	GGATTGGGTTGAGGCAGCTATTG
	CD163	Forward	GAATCACAGCATGGCACAGGTC
		Reverse	CACAAGAGGAAGGCAATGAGAAGG
	GAPDH	Forward	GACATGCCGCCTGGAGAAAC
		Reverse	AGCCCAGGATGCCCTTTAGT

### 2.16 Statistical analysis

Statistical analyses were conducted using R software (v 4.2.2) and GraphPad Prism 9. Differences between two groups were assessed using the Wilcoxon rank sum test, with statistical significance defined as *P* < 0.05.

## 3 Results

### 3.1 Acquisition of key module genes

**Figure 1:**
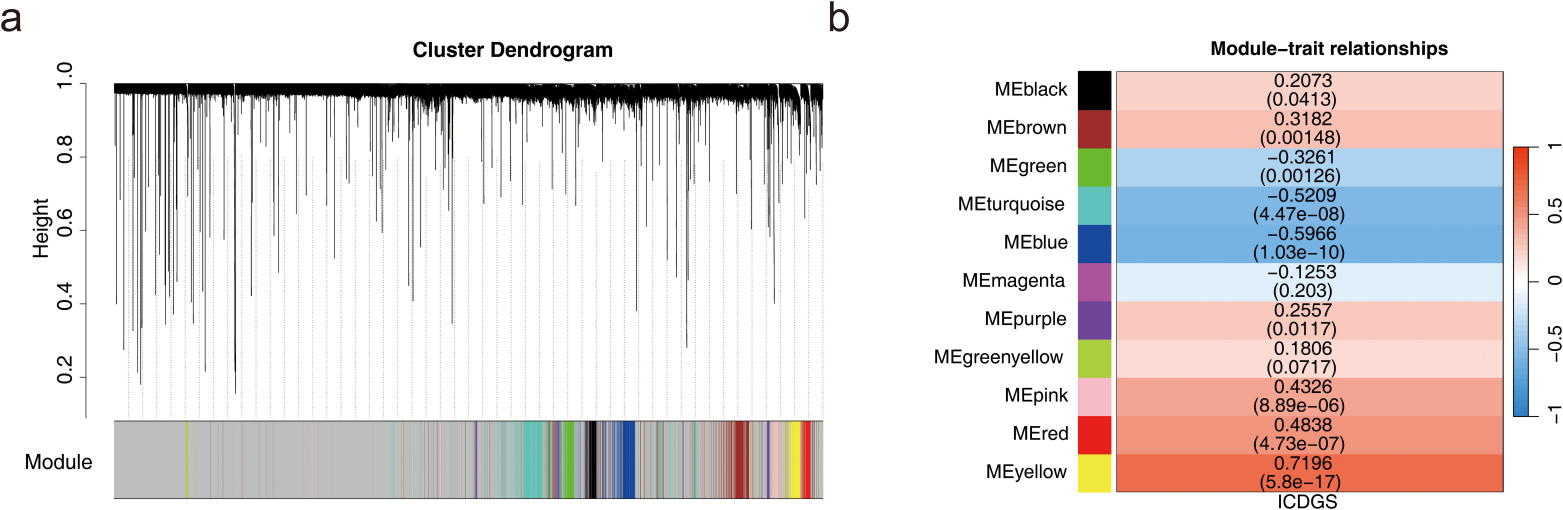
Acquisition of key module genes. **(a)** Co-expression module identification. **(b)** Heatmap showing the correlation between modules and phenotypes.

A total of 441 DEGs were identified, including 236 up-regulated and 205 down-regulated genes in HF (**Additional files 2a-b**). The ssGSEA scores for ICD-related genes significantly differed between HF and normal samples (**Additional file 2c**). In the WGCNA analysis of the GSE57338 dataset, no outlier samples were detected (**Additional file 2d**). The soft threshold was determined to be 7 (**Additional file 2e**). Similar modules were merged from the co-expression matrix, resulting in 11 identified gene modules (excluding the gray module for unclassified genes), with each module represented by a different color (**Figure 1a**). The yellow module (*cor* = 0.72, *P* = 5.8 × 10^–17^) demonstrated the strongest correlation with ICD-related gene ssGSEA scores. Consequently, the 432 genes within the yellow module were designated as key module genes (**Figure 1b**).

### 3.2 Identification and enrichment analysis of candidate genes and PPI

**Figure 2:**
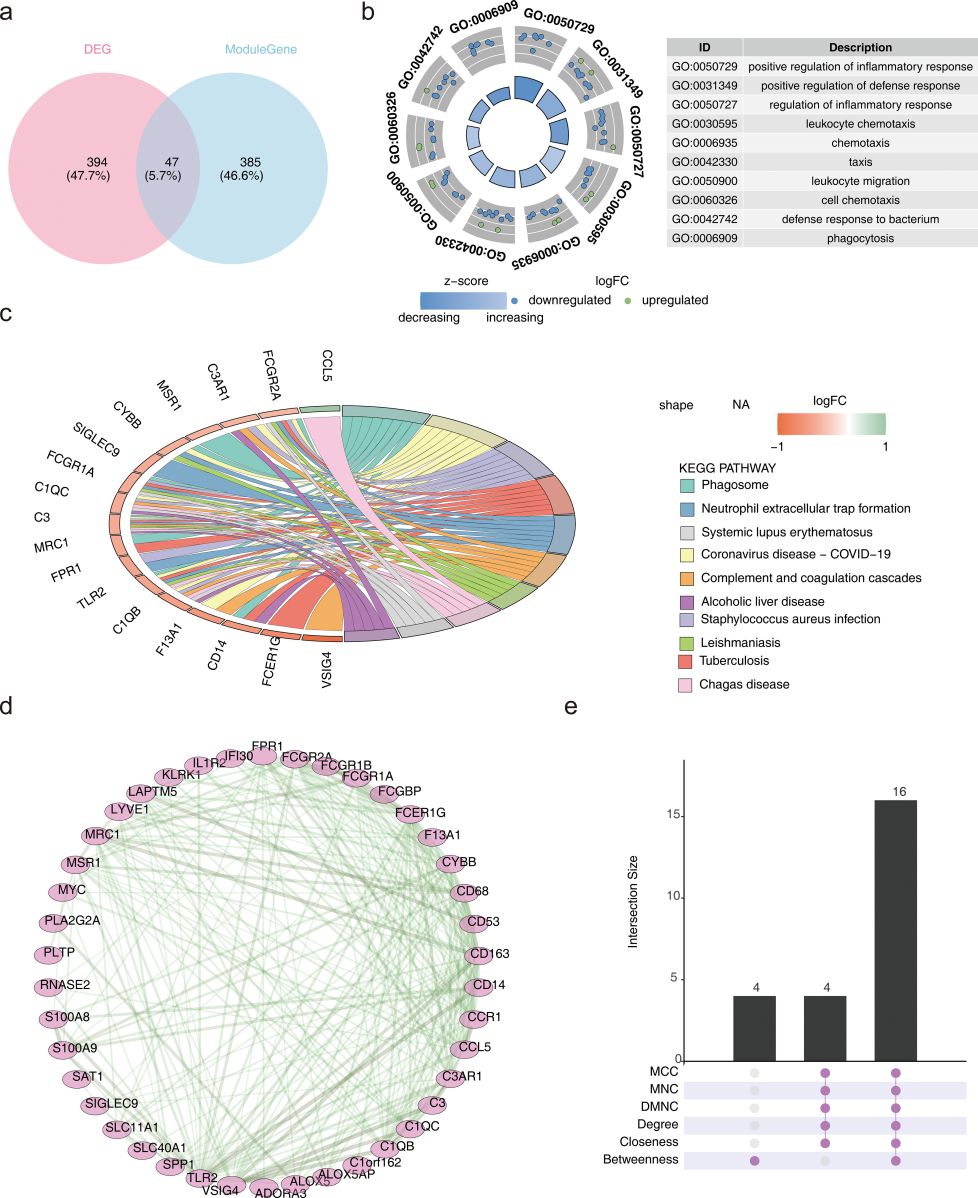
Identification and enrichment analysis of candidate genes and PPI. **(a)** Venn diagram depicting the overlap between differentially expressed genes (DEGs) and key module genes. **(b)** Gene Ontology (GO) enrichment analysis results. **(c)** Kyoto Encyclopedia of Genes and Genomes (KEGG) enrichment analysis results. **(d)** Protein-Protein Interaction (PPI) network. **(e)** Upset plot representing the PPI network.

In this study, 47 candidate genes were identified through the intersection of DEGs and key module genes (**Figure 2a**). The obtained candidate genes were subject to gene ID conversion, though FCGR1B could not be successfully converted. GO enrichment analysis revealed 272 GO terms, comprising 224 biological processes (BP), 24 cellular components (CC), and 24 molecular functions (MF) (*P* < 0.05) (**Figure 2b**). The candidate genes were significantly enriched in pathways such as the positive regulation of inflammatory response, secretory granule membrane, and RAGE receptor binding. Additionally, the candidate genes were enriched in 26 KEGG pathways (*P* < 0.05), including staphylococcus aureus infection, phagosome, and neutrophil extracellular trap formation (**Figure 2c**). These results implied that candidate genes may play important roles in antimicrobial immunity, inflammatory response and cellular damage repair.

The candidate genes were further subjected to PPI network construction, resulting in 42 genes, such as TLR2, FPR1, and MRC1, and 240 gene-to-gene pairs, including TLR2-CD163 and VSIG4-CD14 (**Figure 2d**).

To optimize the screening of candidate genes, the genes were ranked using different algorithms. The top 20 genes from each algorithm were extracted, and the intersection of these top 20 genes was taken. Finally, 16 genes were identified as the candidate key genes for further analysis (**Figure 2e**).

### 3.3 Machine learning for candidate biomarker screening

**Figure 3:**
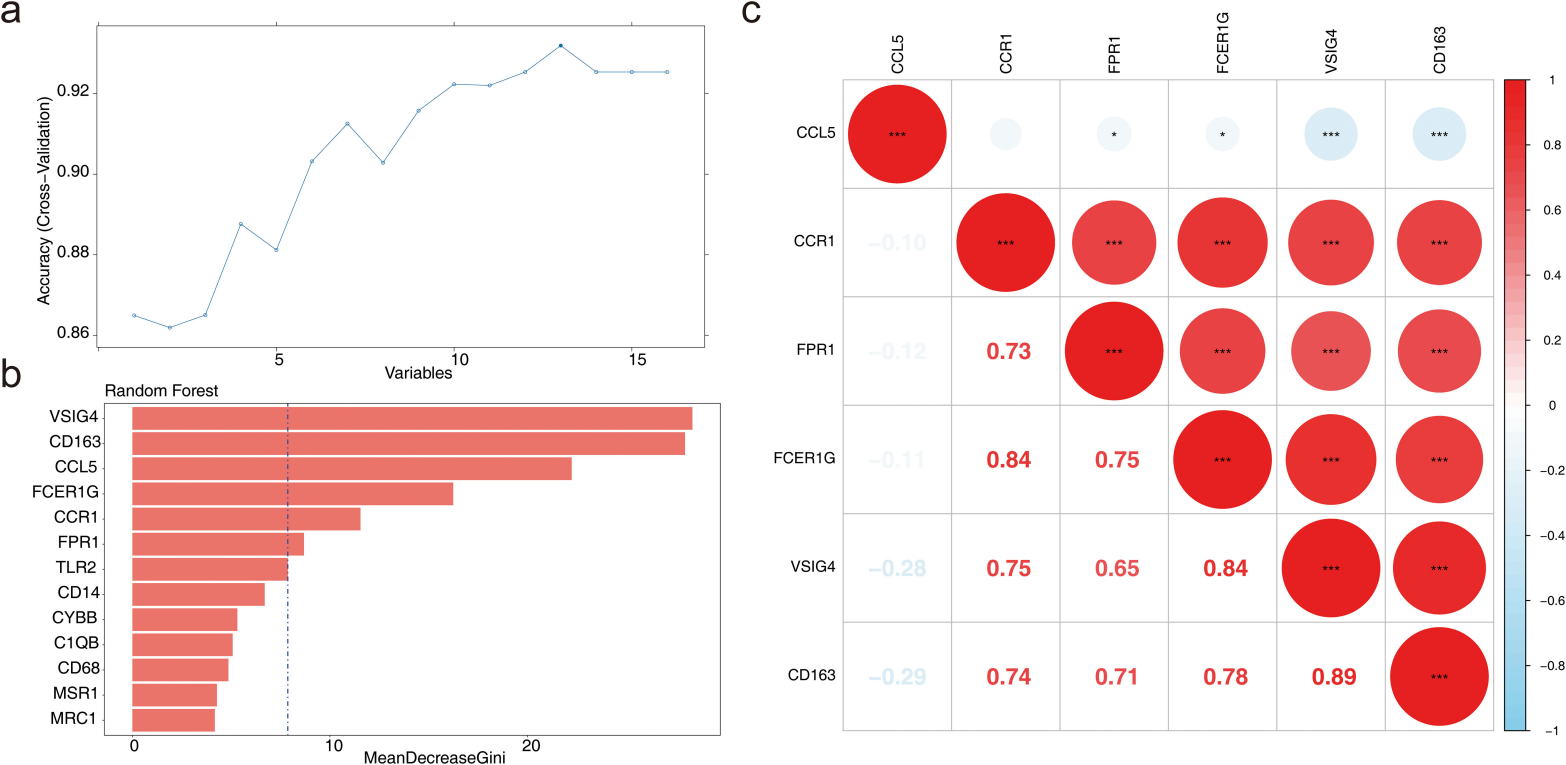
Machine learning for candidate biomarker screening. **(a)** Results of the Support Vector Machine-Recursive Feature Elimination (SVM-RFE) model. **(b)** Bar chart depicting the MeanDecreaseGini scores for candidate genes. **(c)** Correlation analysis of candidate biomarkers. *P < 0.05, ***P < 0.001.

Based on the sample grouping information from GSE57338, the SVM-RFE algorithm was applied for screening, resulting in 13 feature genes: CD163, VSIG4, FCER1G, CCR1, CCL5, FPR1, TLR2, C1QB, CD14, MSR1, CD68, MRC1, and CYBB (**Figure 3a**). The MeanDecreaseGini values for each feature gene ranged from 0 to 30, with notable differences observed between the genes (**Figure 3b**). By calculating the median of the MeanDecreaseGini values, six genes greater than the median were selected as candidate biomarkers: CD163, VSIG4, FCER1G, CCR1, CCL5, and FPR1. Among these, CCL5 showed a negative correlation with VSIG4 and CD163, while the remaining five genes exhibited positive correlations with each other (*P* < 0.01) (**Figure 3c**). The correlation between these genes suggested that they may work in concert at different stages of the immune response or in different types of immune cells.

### 3.4 Diagnosis and evaluation of biomarkers

**Figure 4:**
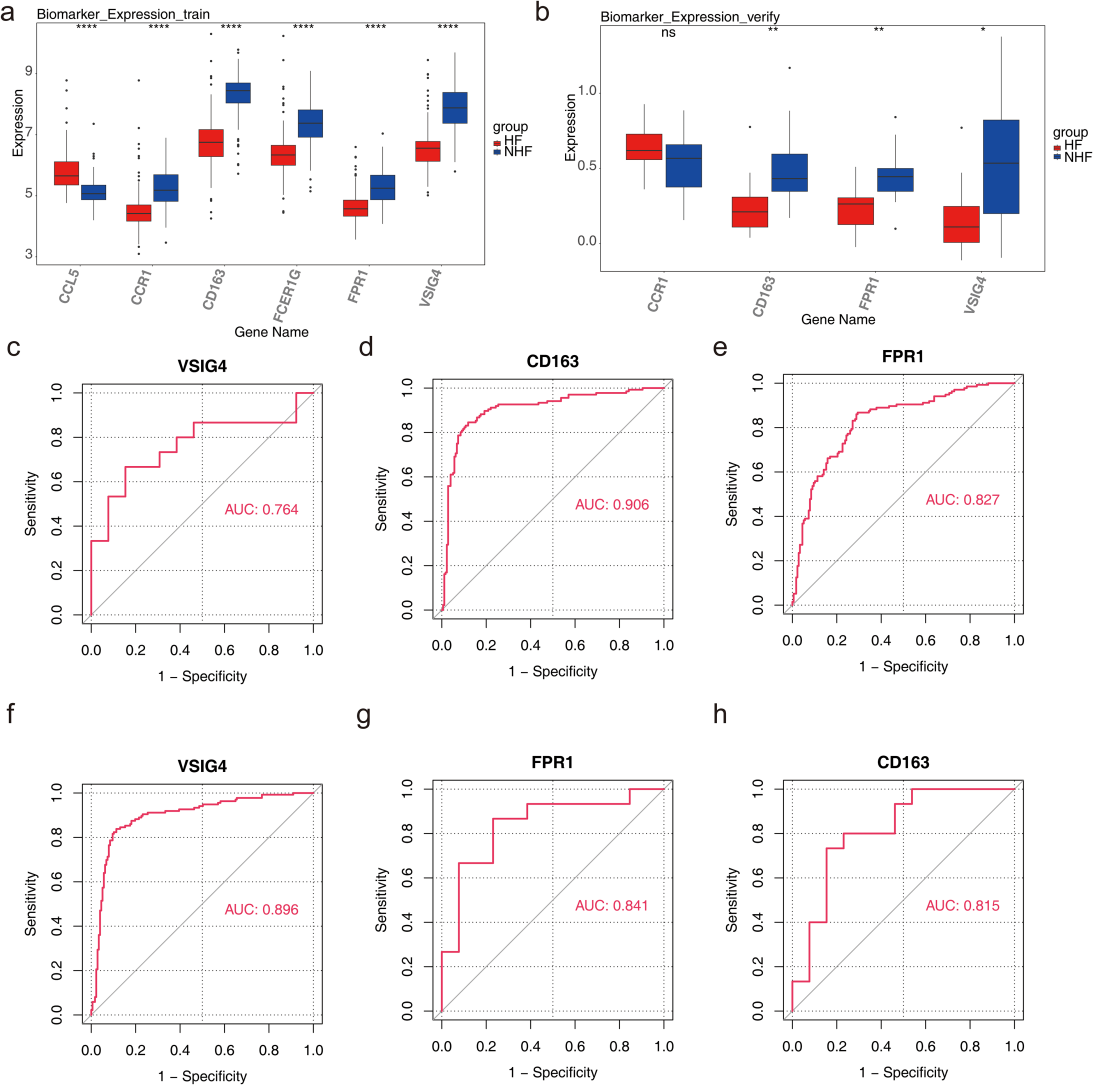
Diagnosis and evaluation of biomarkers. **(a)** Expression levels of candidate genes in the training set, with the horizontal axis representing genes and the vertical axis indicating gene expression levels (Wlicoxon rank sum test, ****P < 0.0001). **(b)** Expression levels of candidate genes in the validation set, with similar axis labels and significance markers (Wlicoxon rank sum test, *P < 0.05, **P < 0.01, ns: *P* > 0.05). **(c)** ROC curve analysis of the VSIG4 biomarker in the validation set. **(d)** ROC curve analysis of the CD163 biomarker in the training set. **(e)** ROC curve analysis of the FPR1 biomarker in the training set. **(f)** ROC curve analysis of the VSIG4 biomarker in the training set. **(g)** ROC curve analysis of the FPR1 biomarker in the validation set. **(h)** ROC curve analysis of the CD163 biomarker in the validation set.

In GSE57338, the six candidate biomarkers demonstrated significant differences between HF and normal samples (*P* < 0.05), with CD163, FPR1, and VSIG4 showing decreased expression in HF samples (**Figure 4a**). In GSE3586, only CD163, VSIG4, CCR1, and FPR1 were expressed, with CD163, FPR1, and VSIG4 levels significantly reduced in HF samples, consistent with the expression patterns observed in GSE57338 (**Figure 4b**). Consequently, CD163, FPR1, and VSIG4 were selected for ROC analysis, which revealed that the AUC for all three biomarkers exceeded 0.7 in both datasets, confirming their potential as HF biomarkers (**Figures 4c-h**). Next, the expression analysis of CD163, FPR1, and VSIG4 in the GSE5406 dataset showed that all three were significantly under-expressed in the HF group compared to the normal group (**Additional file 3**). The expression patterns were consistent with those in the GSE57338 and GSE3586 datasets.

The nomogram model demonstrated that these three biomarkers could accurately predict the risk of HF occurrence. ROC analysis of the nomogram yielded an AUC of 0.913, indicating that the predictive accuracy of the nomogram model was significantly superior to single-gene predictions (**Additional file 4**). It also suggested that the onset and progression of HF may involve complex interactions of multiple genes or biological pathways.

### 3.5 Functional analysis of biomarkers

**Figure 5:**
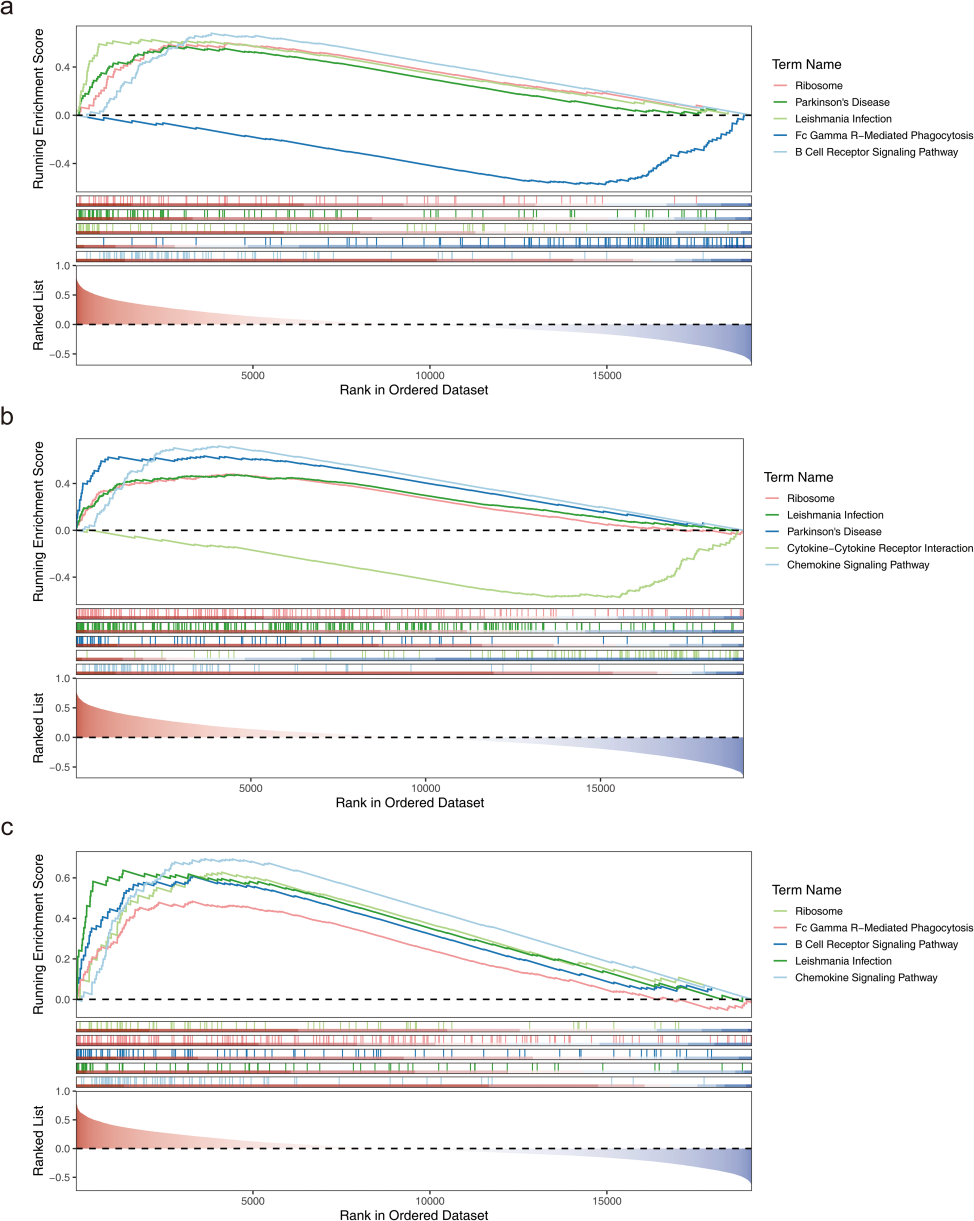
Functional analysis of biomarkers. **(a)** GSEA enrichment analysis of the CD163 gene. **(b)** GSEA enrichment analysis of the FPR1 gene. **(c)** GSEA enrichment analysis of the VSIG4 gene.

Further analysis of the signaling pathways involving CD163, FPR1, and VSIG4 revealed that CD163 was enriched in 76 pathways, including ribosome, Parkinson’s disease, leishmania infection, Fc gamma R-mediated phagocytosis, and B cell receptor signaling (**Figure 5a**). FPR1 was enriched in 79 pathways, including ribosome, leishmania infection, Parkinson’s disease, cytokine-cytokine receptor interaction, and chemokine signaling (**Figure 5b**). VSIG4 was enriched in 85 pathways, including ribosome, Fc gamma R-mediated phagocytosis, B cell receptor signaling, leishmania infection, and chemokine signaling (**Figure 5c**). Notably, all three biomarkers were enriched in pathways related to ribosome function, immune cells, and immune factors. These findings provided a basis for further investigation of the potential applications of biomarkers in immunomodulation, disease diagnosis and therapy.

### 3.6 Analysis of immune cell infiltration

**Figure 6:**
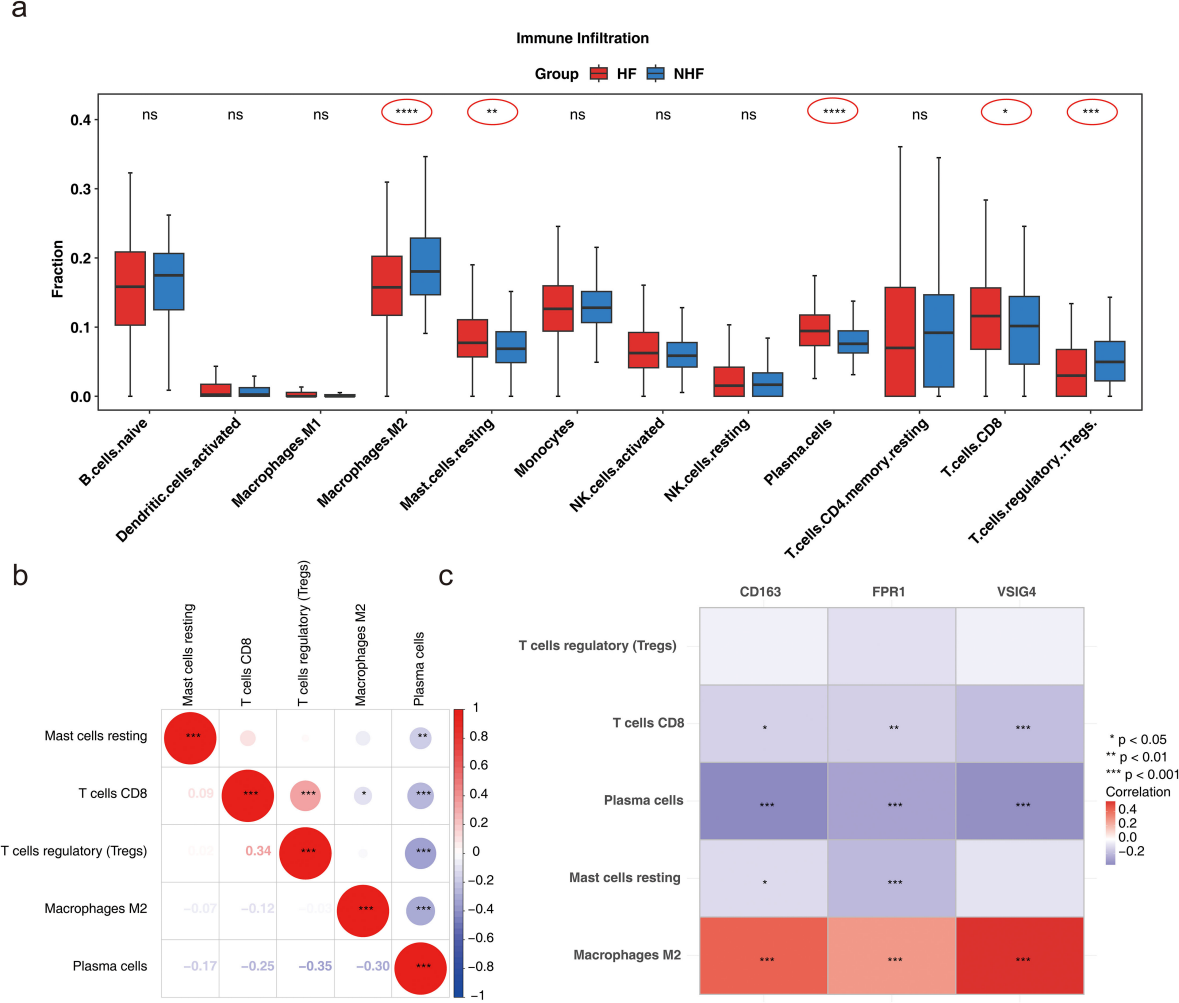
Analysis of immune cell infiltration. **(a)** Box plot illustrating immune cell infiltration differences (Wlicoxon rank sum test, *P < 0.05, **P < 0.01, ***P < 0.001, ****P < 0.0001, ns: *P* > 0.05). **(b)** Correlation of differential immune cell types (*P < 0.05, **P < 0.01, ***P < 0.001). **(c)** Correlation between biomarkers and differential immune cells (*P < 0.05, **P < 0.01, ***P < 0.001).

To further explore immune status differences between HF and normal samples, immune infiltration analysis was performed on GSE57338 samples, revealing differences in the abundance of 22 immune cell types between samples from patients with HF and normal samples (**Additional file 5**). Immune cells with a result of 0 in 30% of the samples were excluded, leaving 12 immune cell types for subsequent analysis. Five immune cell types showed significant differences between the groups: M2 macrophages, resting mast cells, plasma cells, CD8^+^ T cells, and T regulatory cells (Tregs) (*P* < 0.05) (**Figure 6a**). Correlation analysis among these five immune cell types revealed a strong positive correlation between CD8^+^ T cells and Tregs, while plasma cells exhibited negative correlations with Tregs, CD8^+^ T cells, M2 macrophages, and resting mast cells (|cor| > 0.3, *P* < 0.05) (**Figure 6b**). The correlation heatmap between biomarkers and the five immune cell types showed that VSIG4 had a strong positive correlation with M2 macrophages, and M2 macrophages positively correlated with CD163 and FPR1. In contrast, CD8^+^ T cells and plasma cells negatively correlated with CD163, FPR1, and VSIG4, respectively. Resting mast cells demonstrated an inverse correlation with CD163 and FPR1 (*|cor|* > 0.3, *P* < 0.05) (**Figure 6c**). The above results suggested that biomarkers may be involved in disease onset and progression by modulating immune responses and cellular functions.

### 3.7 Molecular regulatory network and drug prediction

**Figure 7:**
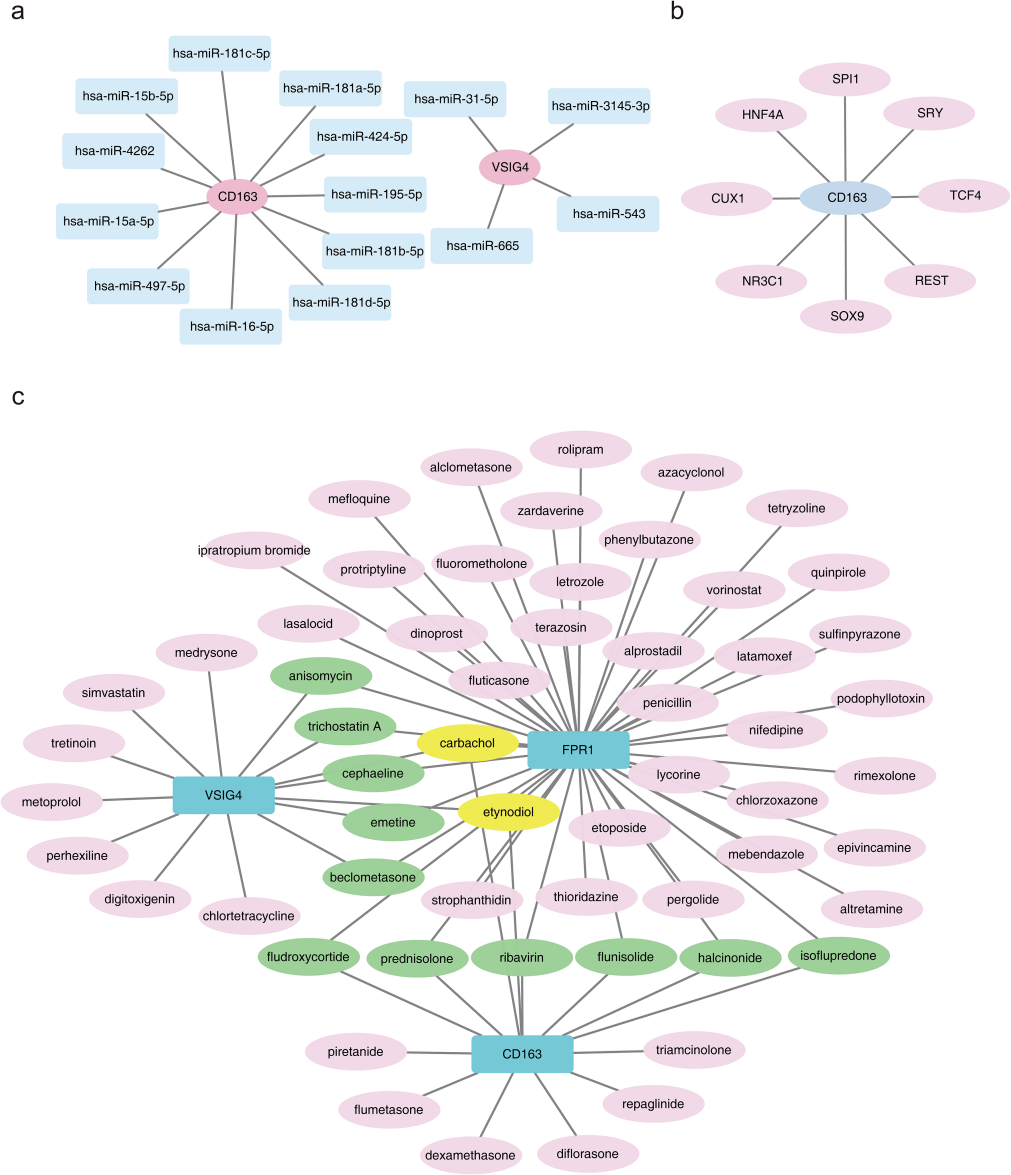
Molecular regulatory network and drug prediction. **(a)** Regulatory network between the CD163 gene and miRNAs, where pink nodes represent biomarkers and blue nodes represent interacting miRNAs. **(b)** Regulatory network between the CD163 gene and transcription factors (TFs). **(c)** Diagram of drug prediction for biomarkers.

Prediction of miRNA interactions with the three biomarkers revealed that VSIG4 was regulated by four miRNAs, including hsa-miR-665; CD163 was regulated by 11 miRNAs, including hsa-miR-4262; while no miRNA regulatory relationships were found for FPR1 (**Figure 7a**). TFs regulating the biomarkers were also analyzed, revealing that no TFs regulated VSIG4 or FPR1, but eight TFs, including SOX9, were found to regulate CD163 (**Figure 7b**). These findings provided important clues for further understanding of immune markers and their regulatory networks in HF.

A total of 74 biomarker-drug/compound relationships were identified. The network analysis suggested that carbachol and etynodiol may have potential effects on all three biomarkers. Additionally, six compounds were shared between CD163 and FPR1—prednisolone, flunisolide, fludroxycortide, halcinonide, ribavirin, and isoflupredone—while five compounds were shared between FPR1 and VSIG4, including anisomycin, trichostatin A, cephaeline, emetine, and beclometasone (**Figure 7c**). By understanding the role of these drugs in regulating the expression of immune markers, more effective therapeutic strategies may be developed in the future.

### 3.8 Single-cell RNA sequencing analysis

**Figure 8:**
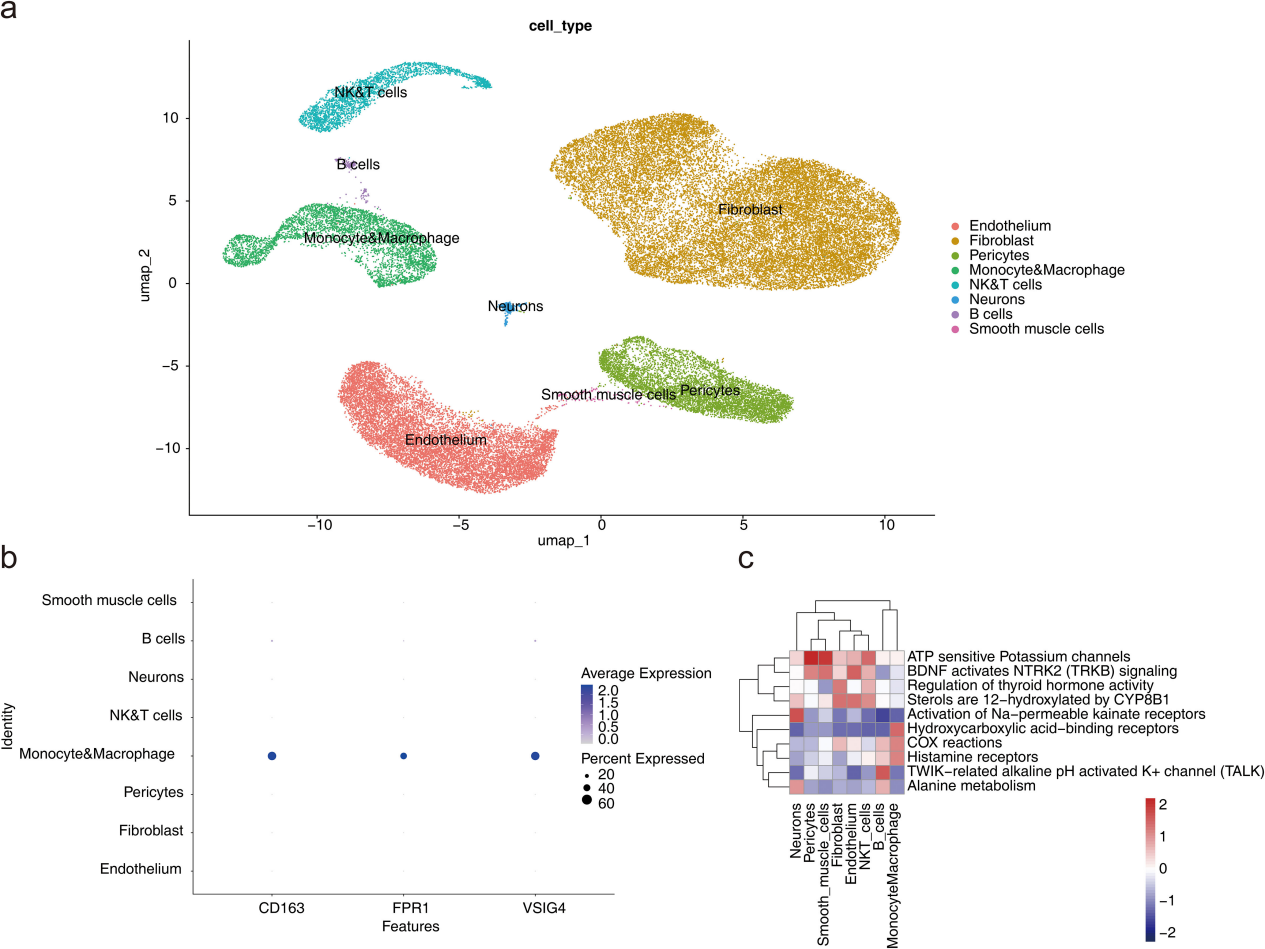
Single-cell RNA sequencing analysis. **(a)** UMAP plot for different cell types. **(b)** Expression profile plot of biomarkers. **(c)** Pathway enrichment analysis of cell subtypes.

Following quality control, 23,963 genes and 49,042 cells were identified (**Additional file 6**). The top 2000 HVGs were selected, and the 10 genes exhibiting the greatest variation were identified (**Additional file 7a**). PCA was performed on the selected HVGs, and the top 10 principal components (PCs) were chosen for further analysis (*P* < 0.05) (**Additional files 7b-c**). UMAP clustering analysis was conducted prior to cell annotation, resulting in the identification of 14 distinct cell clusters (**Additional file 7d**). Nine cell types and their corresponding markers were extracted for annotation based on the reference (12). Subsequently, cell annotation revealed eight distinct cell types: endothelium, fibroblasts, pericytes, monocytes and macrophages, natural killer and T lymphocytes (NK&T cells), neurons, B cells, and smooth muscle cells (**Figure 8a**; **Additional file 8**). Monocytes and macrophages expressing all three biomarkers were designated as key cells (**Figure 8b**). To explore the biological pathways and functions of these cell subtypes in HF development, enrichment analysis revealed that pericytes and smooth muscle cells were significantly associated with ATP-sensitive potassium channels and BDNF activation of NTRK2 (TRKB) signaling, while NK&T cells and B cells were predominantly enriched for activation of Na-permeable kainate receptors and hydroxycarboxylic acid-binding receptors (**Figure 8c**). The above results implied that these cell types act synergistically through multiple mechanisms and may provide new targets and ideas for the treatment of HF.

**Figure 9:**
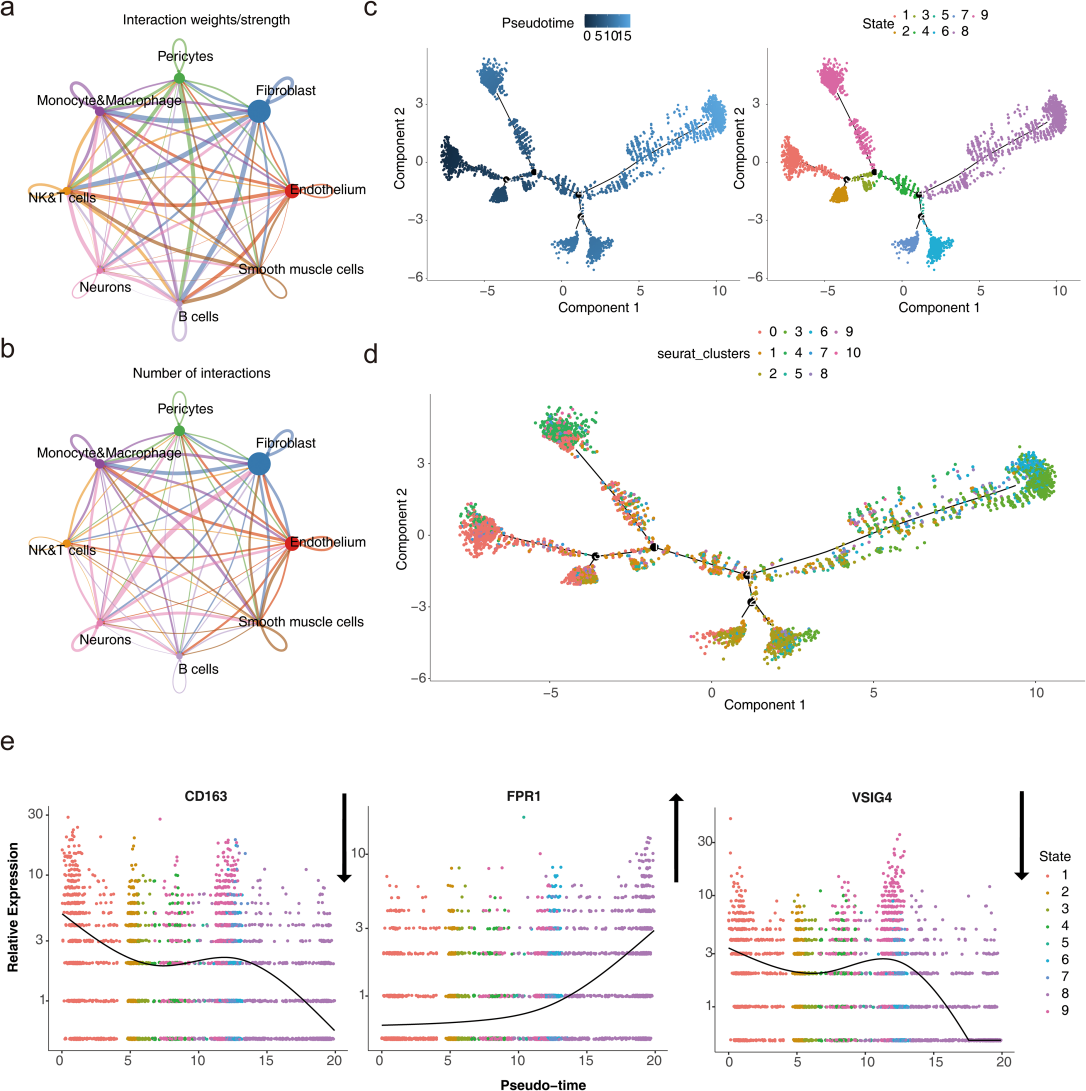
Cell subtype communication analysis. **(a-b)** Diagrams of cell communication results. **(c)** Results of pseudotime and state analysis in single-cell pseudotemporal analysis. Darker colors indicate the most advanced state of development, while lighter colors indicate more mature development. The cells were divided into 9 different periods according to their developmental state. **(d)** Cell pseudotime analysis of Seurat clusters. Different cell clusters presented different positions at various nodes of the developmental trajectory. **(e)** Dynamic atlas of biomarkers.

Analysis of cell communication between the eight cell types showed that fibroblasts and neurons exhibited the highest number of ligand-receptor pairs, indicating the strongest interaction between these two cell types. Fibroblasts also demonstrated a higher probability of communication with monocytes and macrophages, NK&T cells, and B cells (**Figures 9a, b**; a: plot of probability of cellular communication, b: plot of number of cellular communications). The high-frequency interaction of fibroblasts with these immune cells suggested that they may play an important role in tissue repair and remodeling in immune responses, and inflammation.

Monocytes and macrophages were projected onto a root with 9 branches, traversing 9 nodes along their developmental trajectory. Clusters 0 and 3 marked the initial stages of monocyte and macrophage development, while clusters 4 and 6 were primarily located at the final stages of cellular differentiation (**Figures 9c, d**). This dynamic developmental trajectory may reflected how immune cells progressively differentiate and regulate their functions in the body according to different needs.

Given the specific expression of the biomarkers in monocytes and macrophages, the gene expression of the three biomarkers was analyzed across the pseudo-time series. The expression of CD163 showed a decreasing trend over time, with slight increases at certain nodes of the developmental cycle, but overall, the expression in the cells declined. In contrast, FPR1 exhibited an upward trend, indicating its potential significant role in cellular development and differentiation. The expression pattern of VSIG4 mirrored that of CD163 (**Figure 9e**). This expression pattern suggested that their immunosuppressive or reparative functions may be gradually replaced by other functions.

To further explore the biomarker expression of monocyte and macrophage subpopulations at different stages of differentiation, 13 cells were first clustered and annotated into 5 subpopulations based on marker genes (**Table 2**; **Additional file 9a-c**). Subsequently, the five cell subpopulations were analyzed in a proposed time series. As shown in **Additional file 9d**, cells gradually differentiated over time, with darker blue representing earlier differentiation. Each cell subpopulation mapped to a different differentiation time and corresponded to a different differentiation state, with darker red indicating the earliest type of differentiation. As cells differentiated, the expression of CD163 and VSIG4 in key cell subpopulations gradually increased, while the expression of FPR1 slowly decreased (**Additional file 9e**). Next, stacked plots of cell subpopulations in different differentiation states (**Additional file 9f**) showed that M1 macrophages were distributed in all differentiation states, especially more in state 2 and state 5; Intermediate monocytes were distributed only in state 1; and Non-classical monocytes were mainly distributed in state 1 and state 3; M2 macrophages were concentrated in state 3 and state 4 in the later stages of differentiation; Classical monocytes were found mainly in state 1 and state 5. Subsequently, the proportions of cell subtypes under different groupings were visualized (**Additional file 10a**). By comparing NOS2, TNF, ARG1 and MRC1 gene expression in Monocyte&Macrophage between HF and control samples, TNF and MRC1 were found to be significantly different between the two groups (**Additional files 10b-e**). This provided important clues to a deeper understanding of the function of monocytes and macrophages and their role in disease.

**Table 2:** Marker gene annotation information for key cell subpopulations.

TNF	M1 macrophage
MERTK, CD163, STAB1, MRC1	M2 macrophage
BASP1, CXCL8, GPR183	Classical monocyte
FCN1	Non-classical monocyte
FCGR3A	Intermediate monocyte

### 3.9 Clinical and animal validation of Hub genes

**Figure 10:**
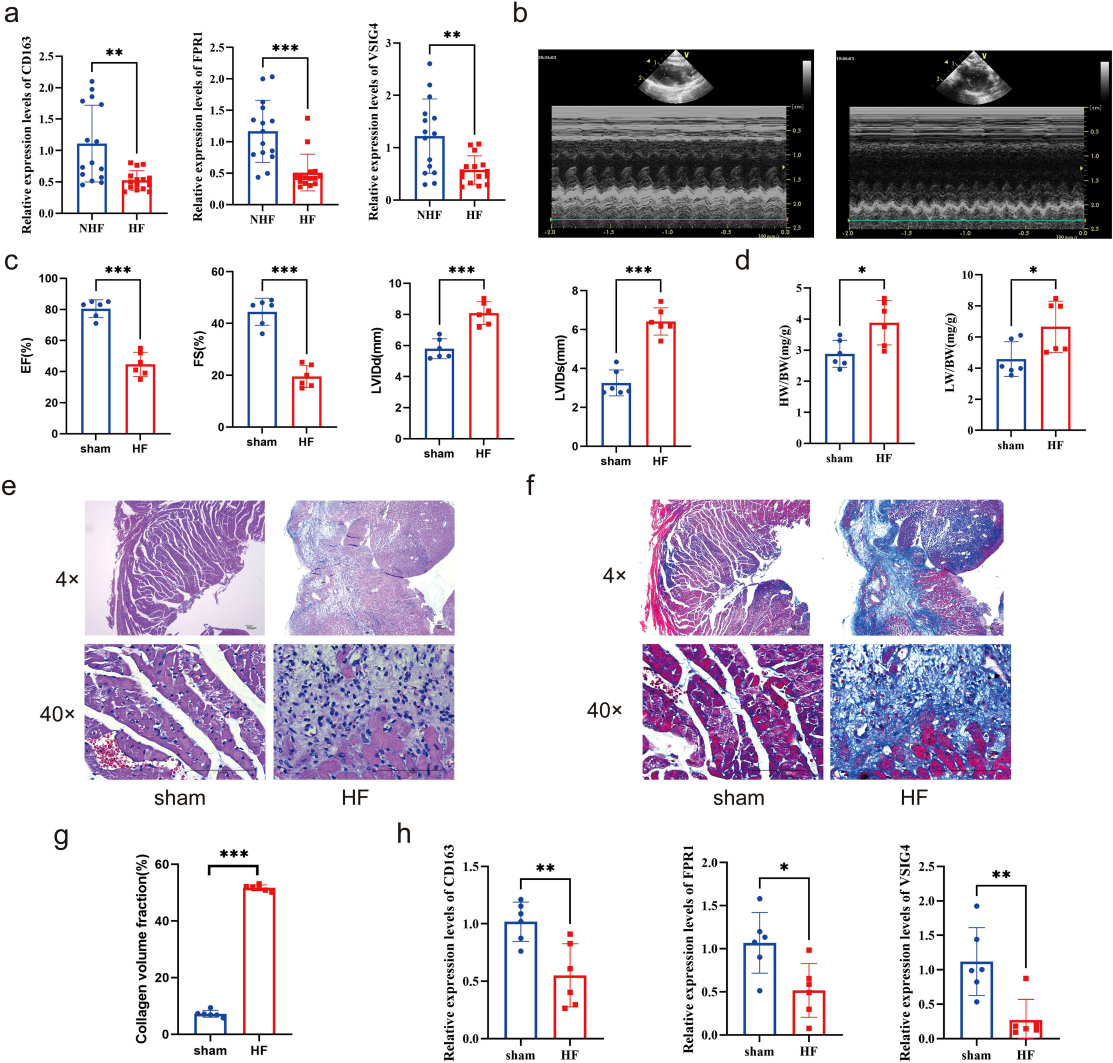
Clinical and animal validation of hub genes. **(a)** Expression of CD163, FPR1, and VSIG4 in peripheral blood mononuclear cells of patients with HF and NHF individuals (Unpaired t test, **P < 0.01, ***P < 0.001). **(b-c)** Echocardiograms of the HF rat model and sham group (Unpaired t test, ***P < 0.001). **(d)** The ratios of heart weight to body weight and lung weight to tibia length in rat model (Unpaired t test, *P < 0.05). **(e-f)** HE and Masson staining of rat hearts. **(g)** Collagen volume fraction(%) calculated by Masson staining (Unpaired t test, ***P < 0.001). **(h)** Expression of CD163, FPR1, and VSIG4 in the hearts of HF and sham groups (Unpaired t test, *P < 0.05, **P < 0.01).

To validate the expression levels of ICD-related hub genes in HF, PBMCs were extracted from 15 clinical patients with HF and controls for RT-qPCR analysis. Results revealed significant down-regulation of CD163, FPR1, and VSIG4 in patients with HF (**Figure 10a**). Further investigation was conducted in heart tissues using the HF rat model. Echocardiography showed reduced left ventricular ejection fraction (LVEF) and left ventricular fractional shortening index (LVFS), alongside increased left ventricular end-systolic diameter (LVIDs) and left ventricular end-diastolic diameter (LVIDd) in HF rats (**Figures 10b-c**). The ratios of heart weight to body weight and lung weight to tibia length were significantly elevated (**Figure 10d**). HE staining revealed prominent cardiomyocyte hypertrophy, with inflammatory cell infiltration in the HF group (**Figure 10e**). Masson staining indicated severe fibrosis in the HF group (**Figure 10f**), and the difference in fibrosis between the two groups was significant (**Figure 10g**). Cardiac tissue RT-qPCR results confirmed that CD163, FPR1, and VSIG4 were significantly down-regulated in HF rats (**Figure 10h**). The results of immunohistochemistry showed that the expression of CD163^+^ cells was decreased in the myocardial tissue of HF mice (**Additional file 11**). These results suggested that down-regulation of CD163, FPR1, and VSIG4 expression in HF patients and HF rat models may be closely associated with dysregulation of the immune system, decreased cardiac function, and tissue damage.

## 4 Discussion

Cardiac immunology has recently emerged as a focal area of research. While some aspects of immune regulation in HF are understood, many questions remain to be addressed. ICD is a form of programmed cell death induced by antigens and adjuvants, triggering downstream immune responses. However, the role and mechanisms of ICD in HF pathophysiology remain unclear. In this study, three ICD-related biomarkers—CD163, FPR1, and VSIG4—were identified in patients with HF using transcriptomic and single-cell dataset analyses (**Additional file 12**). Previous studies have shown that these three genes, as combined markers, may act synergistically to affect the occurrence and development of HF and non-alcoholic fatty liver disease by regulating mechanisms such as immune response and monocyte migration. In addition, their association with natural killer (NK) cells and macrophages was also found, further supporting their important role in the immune response (39).

Single-cell sequencing data in this study were obtained from the research by Koenig et al. (12). Unlike the study by Koenig, our work systematically integrated multiomics analyses (including transcriptomes and single-cell sequencing), machine-learning approaches (e.g., SVM-RFE and random forests), and immune infiltration assessments, which were not comprehensively combined in their study. Furthermore, this study identified the association of CD163, FPR1, and VSIG4 with ICD, a connection that Koenig et al. did not investigate. Specifically, during ICD, certain molecules, especially ANXA1, may enhance local inflammatory responses by binding to FPR1 receptors and activating macrophages and monocytes. At the same time, the activation of fibroblasts may promote vascular wall structural changes and fibrosis (40). Therefore, targeting FPR1 or ICD-related pathways may be a potential strategy for the treatment of ascending aortic aneurysm. When tumor cells develop ICD through radiotherapy or other therapeutic modalities, macrophages recognize tumor cell death signals through CD163 receptors. CD163^+^ macrophages are normally in an immunosuppressive state and help tumors evade immune surveillance by promoting Treg cell infiltration and inhibiting effector T cell function (41). In addition, carbon ion radiotherapy has been shown to effectively reduce fiber deposition in scar tissue by inducing ICD of fibroblasts, slowing their proliferation and promoting their death (42). Another study pointed out that ICD may affect cancer-associated fibroblasts by regulating immune responses, thereby altering tumor progression and patient survival prognosis. Although no association between ICD and macrophages or fibroblasts has been found in HF, these immune cells may affect the occurrence and development of HF through the ICD process. Additionally, potential therapeutic targets were proposed via drug prediction, such as carbachol and etynodiol, which target all three biomarkers. Collectively, this study not only extends the findings of Koenig et al. but also offers novel insights and references for future research in HF.

CD163 (Cluster of Differentiation 163), a 130 kDa cell surface glycoprotein, is predominantly expressed on monocytes and macrophages. It plays significant roles in metabolic diseases and immune regulation and is considered a promising target for drug development (43, 44). Soluble CD163 (sCD163) is a soluble inflammatory mediator produced through the enzymatic hydrolysis of CD163 (45). CD163 expression tends to be low in conditions such as non-alcoholic fatty liver (39, 46) and ischemic cardiomyopathy (47), whereas sCD163 tends to be elevated in hypertension (48) and diabetes (49, 50). Additionally, sCD163 has been linked to increased cardiovascular mortality in diabetic patients. In HF, CD163 expression is down-regulated in cardiac tissues (39, 51), consistent with both bioinformatics and experimental findings in this study. CD163 expression in cardiac tissue is also associated with hyperlipidemia (52) and cellular stemness (51). Moreover, sCD163 is highly expressed in the blood of patients with HF (53), though the mechanisms driving this increase remain under investigation. Some studies suggest that sCD163 levels are influenced by left ventricular diastolic volume (53), while others have linked sCD163 to monocyte activation, particularly activation related to the M2 phenotype (54), which warrants further exploration. In addition, research has demonstrated that CD163 serves as a critical link between the immune system, inflammatory response, and cardiovascular disease by not only reflecting the activation of immune cells, particularly macrophages, but also modulating immune responses (54). Furthermore, in another study, CD163, acting as a macrophage marker, was found to play a significant role in regulating inflammation and the tumor microenvironment (44). This study also found a reduction in CD163 expression in macrophages in HF, suggesting that the progression of HF may be linked to decreased CD163 expression in macrophages.

FPR1 (Formyl Peptide Receptor 1), a key member of the G protein-coupled receptor family, plays a critical role in the inflammatory process and immune cell recruitment. It is highly expressed in macrophages (55) and mediates macrophage chemotactic motility and functional activation by binding to TAFA4 (56, 57). FPR1 is a well-established target for clinical therapeutic drugs, with various agonists and inhibitors developed for its modulation (58). Studies have demonstrated that FPR1 modulates the immune response and repair process of the heart by regulating macrophage activity, and dysregulation of the immune response following cardiac injury may contribute to the development of HF (59). Moreover, FPR1 may mitigate inflammatory responses and facilitate cardiac repair and recovery in HF through the regulation of macrophage function (60). Studies suggest that FPR1 may be a promising drug target for cardiovascular diseases, aiding both diagnosis and treatment (61). It plays a negative regulatory role in myocardial ischemia-reperfusion and coronary atherosclerosis but a positive regulatory role in myocardial infarction. FPR1 contributes to atherosclerotic lesions by modulating the number of blood neutrophils under hypercholesterolemia (62) and exacerbates myocardial cell apoptosis and inflammation during ischemia-reperfusion through the MAPK signaling pathway (63). However, FPR1 activation has been shown to improve left ventricular remodeling after myocardial infarction in mice and rats, potentially by promoting early neutrophil migration and infiltration, thus accelerating wound healing (64). In the present study, decreased expression of FPR1 was observed in PBMCs from patients with HF and in the hearts of HF rats through both bioinformatics and experimental validation. However, no significant difference in FPR1 expression was found in macrophages in HF. Notably, FPR1 expression gradually increased during macrophage differentiation, suggesting its potential as a therapeutic target for HF.

VSIG4 (V-set and immunoglobulin domain containing 4) is a type I transmembrane receptor that inhibits T cell activation and induces the differentiation of regulatory T cells, thus suppressing immune-mediated inflammatory diseases (65). Soluble VSIG4, shed from the surface of macrophages, serves as a biomarker for diseases associated with macrophage activation (66). VSIG4 has a protective role in cardiovascular diseases and can alleviate age-related insulin resistance and hypertension (67). Additionally, research has highlighted that VSIG4, as a critical immune marker, is strongly associated with macrophage function and plays a pivotal role in both the immune response and the diagnosis of HF (39, 68). In myocardial ischemia/reperfusion (I/R) injury, VSIG4 inhibits M1 macrophage polarization by blocking TLR4/NF-κB signaling, thus preventing cardiomyocyte apoptosis (69). However, VSIG4 expression in M2 macrophages promotes fibrosis after acute myocardial infarction, suggesting its potential as an immunomodulatory therapeutic target (70). In HF, VSIG4 expression is significantly down-regulated in patients with right ventricular HF (71), while serum levels of VSIG4 are elevated in patients with left ventricular HF, with high levels correlating with poor prognosis (72). In the present study, VSIG4 expression was decreased in macrophages in HF, and its expression showed a decreasing trend during macrophage differentiation, further suggesting that HF progression may be linked to the expression of VSIG4 in macrophages.

GSEA enrichment analysis reveals that the three biomarkers are significantly enriched in ribosomes. The enhanced translation function of ribosomes is a hallmark of cardiac hypertrophy, and inhibiting ribosomal translation can effectively mitigate hypertrophy (73). However, systemic inhibition of ribosomal translation may cause adverse effects in organs outside the heart. For example, while rapamycin effectively inhibits cardiac hypertrophy, it can lead to severe consequences such as immune suppression (74). Recent studies have identified the cardiac-specific nuclear ribonucleoprotein (RNP)-binding long non-coding RNA (lncRNA) CARDINAL, which alleviates cardiac hypertrophy *in vivo* and *in vitro* by inhibiting the translation of hypertrophy-related proteins (75). In the study by Koji Kasahara et al. (76), FPR1 indirectly influenced ribosomal function through the regulation of ribosomal protein gene expression. Additionally, VSIG4, an up-regulated gene, is linked to ribosome function, implying its potential significance in protein synthesis or cellular function regulation (77). Prior research has demonstrated that CD163 expression correlates with the mTOR signaling pathway (78), which governs translation initiation and ribosome biogenesis (79). The biomarkers identified in this study are all associated with ribosomes, offering a new avenue for basic research. Single-cell analysis highlights the pivotal role of monocytes and macrophages in HF progression, with cardiac macrophages regulating both survival and adaptive remodeling in patients with HF. However, these macrophages are highly infiltrated in the hearts of patients with HF, potentially due to the elevated expression of Ang II, which mobilizes macrophages (80). Macrophages are categorized into M1 and M2 types based on their secreted factors and functions. Promoting the conversion of M1 to M2 macrophages and maintaining a balance between these two subtypes may provide an effective strategy for treating HF (81). It has been demonstrated that sodium-glucose cotransporter 2 (SGLT2) inhibitors can reduce fibrosis markers by promoting M2 macrophage polarization and enhancing angiogenic factors (82), while nicorandil can suppress the production of pro-inflammatory cytokines by inhibiting M1 polarization (83). Furthermore, this study found a positive correlation between the expression levels of these three biomarkers and M2 macrophages, suggesting that targeting these biomarkers to modulate macrophage homeostasis in HF may offer a promising therapeutic strategy.

Cell subtype communication analysis revealed that fibroblasts likely engage in frequent interactions with monocytes, macrophages, NK cells, T cells, and B cells. Previous studies have demonstrated that macrophages influence cardiac function by modulating fibroblast activity and affecting the remodeling and excessive deposition of extracellular matrix (ECM) (84). During cardiac inflammation and remodeling, macrophages and fibroblasts exhibit a close interconnection. Notably, M1 macrophages release pro-inflammatory cytokines, activate fibroblasts, and drive the progression of fibrosis (85). Additionally, research has shown that macrophages interact with TWEAK via the receptor CD163, playing a critical role in cardiac fibrosis and HF (86). VSIG4 promotes cardiac fibrosis repair during acute myocardial infarction (AMI) by regulating M2-type macrophage function and interacting with immune factors such as TGF-β1 and IL-10 (70). Furthermore, the FPR1 receptor is crucial for the aggregation and activation of immune cells, including monocytes and macrophages, which subsequently impacts fibroblast activation and fibrosis, thereby promoting inflammatory and fibrotic responses in the heart and lung (87). Collectively, the intricate crosstalk between immune cells and fibroblasts plays a pivotal role in the pathogenesis of cardiac inflammation and fibrosis, offering potential therapeutic targets and novel strategies for treating cardiac fibrosis.

In this paper, drug prediction was performed based on three biomarkers, and it was found that carbachol and etynodiol may have potential roles for all three biomarkers. Carbachol, a structural analogue of acetylcholine that acts on muscarinic and nicotinic receptors, is used clinically to treat glaucoma (88). Only a few literatures have found that carbachol increases phagocytosis of macrophages *in vitro* (89). Progestin is the first progestin with moderate progestogen activity, and progestin has some effect on macrophages. In a clinical study of adolescent endometriosis, one-year progestin treatment increased the number of CD206^+^ monocytes (P < 0.001) but decreased the number of CD163^+^ monocytes (P = 0.017) (90). The specific effects of the above two drugs on macrophages are still superficial, and the relevant mechanisms are not deeply studied. In addition, the effects of the above two drugs on heart failure are lack of relevant research support and still need to be further explored.

This study has several limitations. First, the dataset is relatively small, necessitating the inclusion of larger, multi-center datasets (e.g. UK Biobank, HF registry study data) for more robust conclusions. Furthermore, validation in human and animal models is preliminary; additional functional experiments, such as gene knockout or overexpression studies, are needed to clarify the roles of these biomarkers in HF progression. Simultaneously, further experimental evidence is required to clarify the relationship between biomarkers and ribosomes. Moreover, existing studies have predominantly focused on monocytes/macrophages, while the interactions with other cell types, such as fibroblasts and cardiomyocytes, remain underexplored. Future investigations could leverage spatial transcriptome technologies, like Visium, to map co-localization regions and deepen our understanding of macrophage-fibroblast interactions. Lastly, the absence of experimental validation for drug predictions restricts their direct clinical application. In subsequent studies, carbachol or etynodiol could be administered in HF rat models to monitor changes in CD163/VSIG4 expression levels, cardiac function parameters, and inflammatory/fibrosis markers. Despite these limitations, the study identifies novel mechanisms underlying HF and highlights potential biomarkers, offering valuable insights for the prevention and treatment of HF and establishing a foundation for future research.

## 5 Conclusions

This study identified three biomarkers—CD163, FPR1, and VSIG4—associated with immunogenic cell death in patients with HF, integrating transcriptomic data with single-cell datasets. The functions and biological pathways of these biomarkers were examined, and the potential links between immunogenic cell death-related genes and HF pathophysiology were explored. Additionally, the expression of these biomarkers was validated in both human and animal models, providing a novel theoretical framework for clinical diagnosis and treatment of HF.

## Data Availability

The original contributions presented in the study are included in the article/**Supplementary Material**. Further inquiries can be directed to the corresponding author.

## Supplementary material

Additional file 1 Thirty-four ICD-related genes.

Additional file 2 Analysis of differential gene expression and sample clustering in HF. **(a)** Volcano plot illustrating differentially expressed genes. **(b)** Heatmap displaying the top 10 differentially expressed genes. **(c)** Comparison of ssGSEA scores between heart failure and normal samples. The horizontal axis represents the ssGSEA score, and the vertical axis represents sample groupings (Wlicoxon rank sum test, ** *P* < 0.01). **(d)** Hierarchical clustering of samples. Each branch in the clustering tree corresponds to a sample, with the vertical coordinate representing the Euclidean distance of sample expression levels. **(e)** Soft-thresholding analysis for network construction.

Additional file 3 Expression analysis of biomarkers in GSE5406 dataset.

Additional file 4 ROC curves of single-gene and nomogram models.

Additional file 5 Stacked column chart depicting differences in immune cell infiltration.

Additional file 6 Quality control results of single-cell sequencing.

Additional file 7 Cellular heterogeneity and dimensionality reduction analysis. **(a)** Diagram for screening highly variable genes. **(b-c)** Principal component analysis (PCA) plots of cells. **(d)** UMAP plot for different cell clusters.

Additional file 8 Bubble chart of cell marker genes.

Additional file 9 Characterization of Monocyte and Macrophage subpopulations and their differentiation dynamics. **(a-b)** Expression levels of Marker genes in different cell populations. **(c)** Annotated cellular map of monocyte and macrophage subpopulations. **(d)** Proposed temporal analysis plot of key cell subpopulations. **(e)** Changes in biomarker expression during cell differentiation. **(f)** The stacked plots of cell subpopulations in different differentiation states.

Additional file 10 TNF and MRC1 were significantly different between HF and control samples. **(a)** Visualization of the proportions of cell subtypes under different groupings. **(b-e)** Expression of NOS2, TNF, ARG1, and MRC1 genes in Monocyte&Macrophage between HF and control samples.

Additional file 11 Expression of CD163^+^macrophages in rat myocardium.

Additional file 12 The flowchart of this research.
