# Supplementary figures and images for: Immunogenic cell death-related biomarkers in heart failure probed by transcriptome and single-cell sequencing
Source: Front Immunol. 2025 Jun 24;16:1560903. doi: 10.3389/fimmu.2025.1560903 (PMC12234543; doi:10.3389/fimmu.2025.1560903)

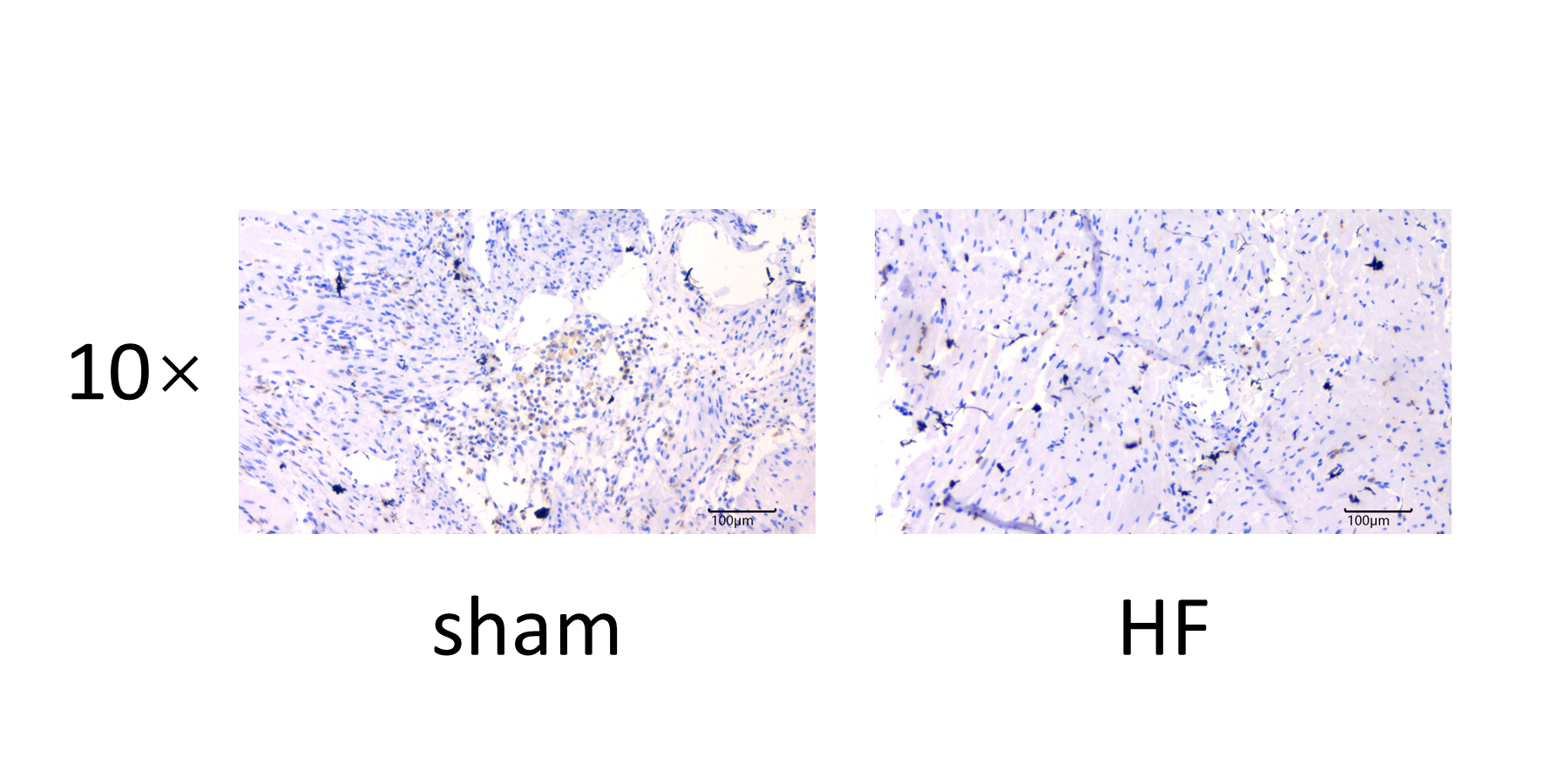

Supplement: Additional file 1 — Thirty-four ICD-related genes. [file DataSheet1.zip › additional file/Additional file 11.tif]

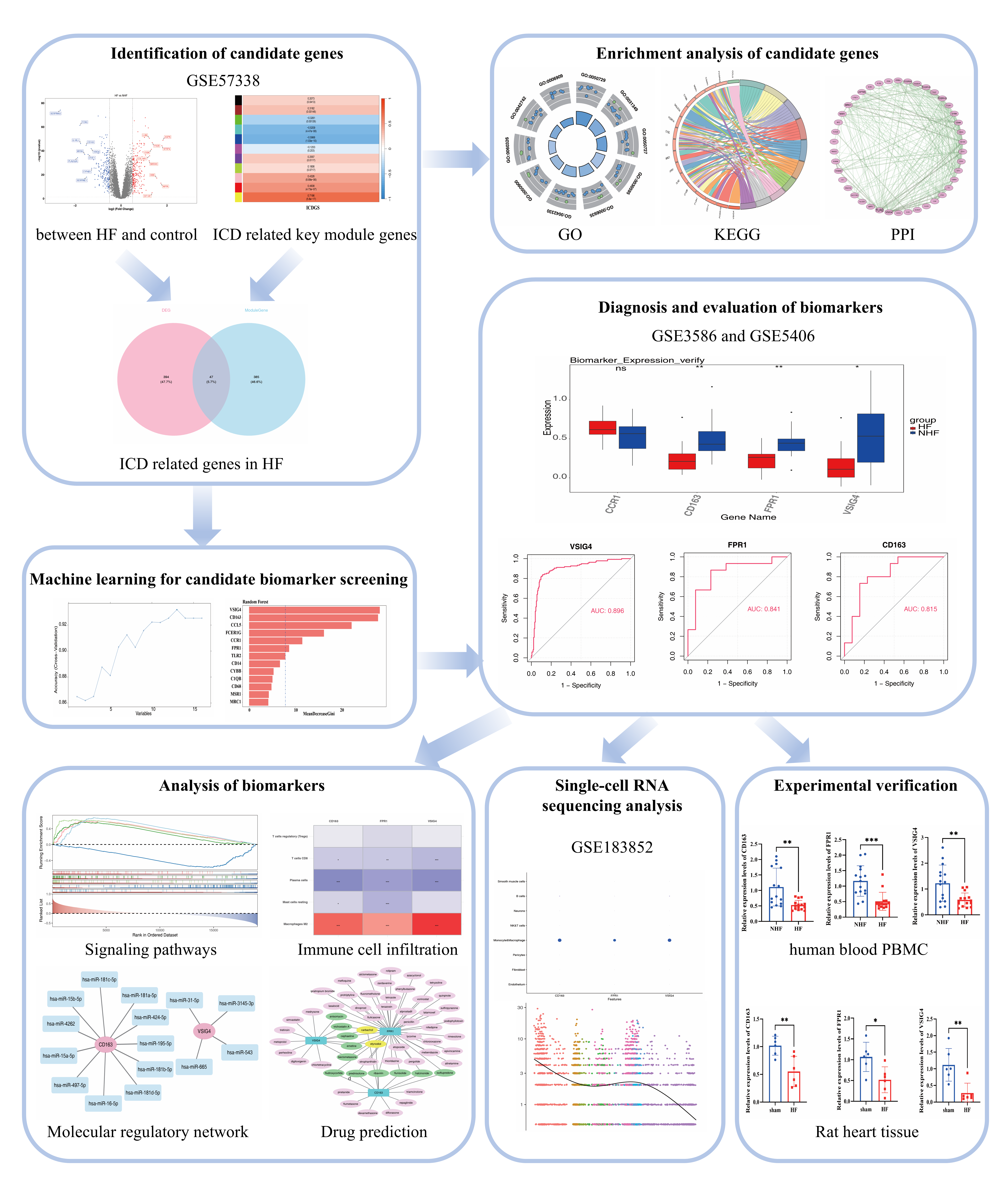

Supplement: Additional file 1 — Thirty-four ICD-related genes. [file DataSheet1.zip › additional file/Additional file 12.tif]

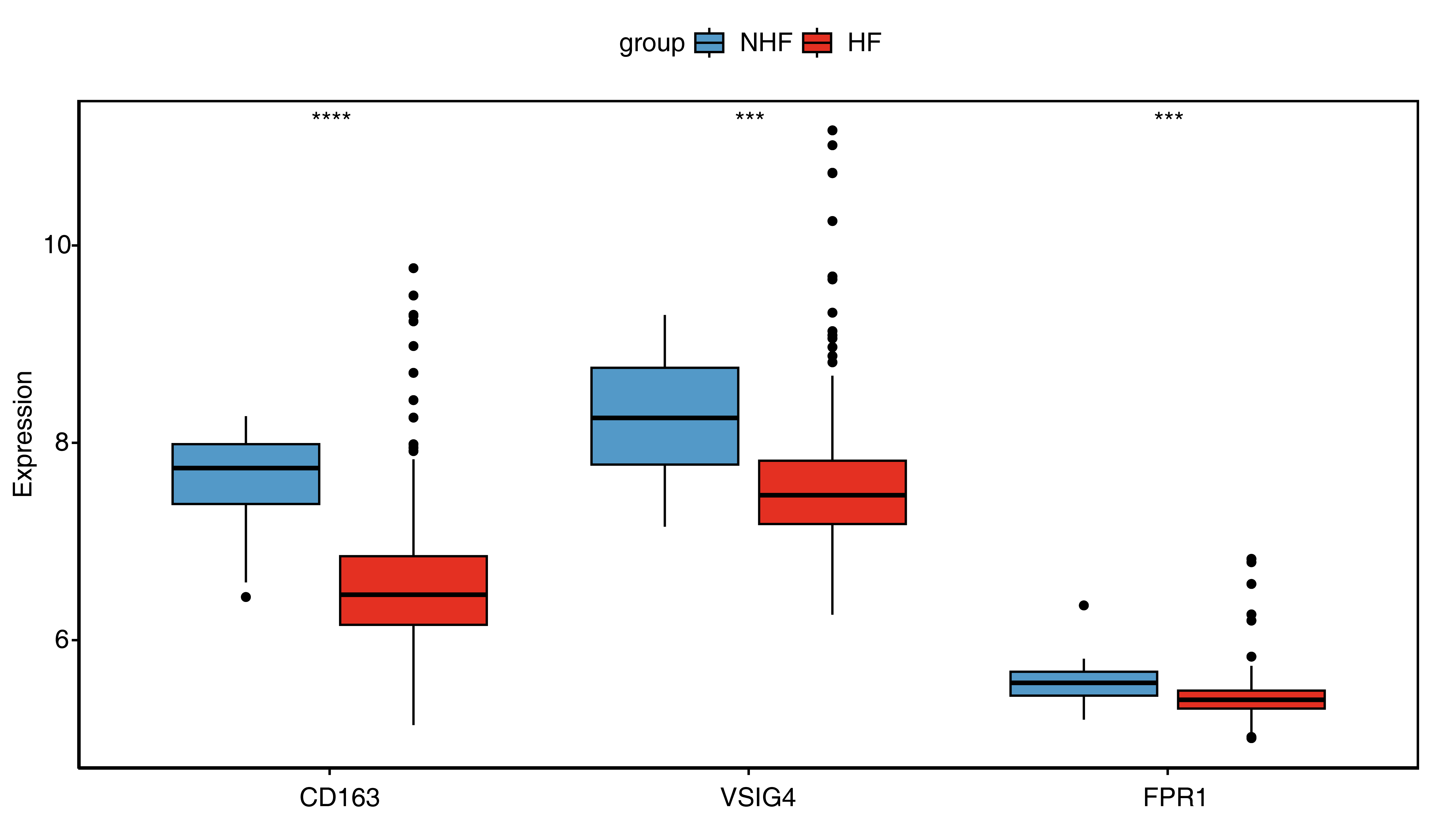

Supplement: Additional file 1 — Thirty-four ICD-related genes. [file DataSheet1.zip › additional file/Additional file 3.tif]
